# Sleep–wake rhythm and its association with lifestyle, health-related quality of life and academic performance among Japanese nursing students: a cross-sectional study

**DOI:** 10.1186/s12912-021-00748-3

**Published:** 2021-11-09

**Authors:** Momoko Kayaba, Toshiko Matsushita, Noriko Katayama, Yuichi Inoue, Taeko Sasai-Sakuma

**Affiliations:** 1 Department of Somnology, Tokyo Medical University, 6-7-1 Nishishinjuku, Shinjuku-ku, Tokyo, 160-0023 Japan; 2 Japan Somnology Center, Institute of Neuropsychiatry, 5-10-10, Yoyogi, Shibuya-ku, Tokyo, 151–0053 Japan; 3 Department of Nursing, Graduate School of Medicine, Yokohama City University, Fukuura 3-9, Yokohama Kanazawa-ku, Kanagawa, 236-0004 Japan; 4 Graduate School of Health Sciences, Shonan University of Medical Sciences, Kamishinano 16-48, Yokohama Totsuka-ku, Kanagawa, 244–0806 Japan; 5 Department of Clinical Laboratory Science, Faculty of Medical Technology, Teikyo University, Kaga 2-11-1, Itabashi-ku, Tokyo, 173-8605 Japan

**Keywords:** Academic performance, Chronobiology phenomena, Sleep disorders, Circadian rhythm, Sleep hygiene, Students, Nursing, Quality of life

## Abstract

**Background:**

Young adults are likely to have activities and go to bed late at night due to their age-dependent delayed endogenous circadian clock. The purpose of the present study was to clarify sleep–wake rhythm and its association with lifestyle, health-related quality of life, and academic performance among nursing students.

**Methods:**

Self-reported questionnaires were distributed to undergraduate nursing students at six universities in Japan. Sleep–wake rhythm was assessed using the morningness-eveningness questionnaire. A quantitative design using the generalized linear mixed effect model was utilized to identify the factors related to the evening type among female nursing students (*n* = 447).

**Results:**

About 18% of the participants were identified as the evening type. Evening type was associated with living alone, part-time job, and club activity. Sleep duration on weekdays was shorter, meal time duration was the shortest, and the percentages of students who skipped meals and those who gained body weight were higher in the evening type than in the morning type and intermediate type. Mental health-related quality of life was lower in the intermediate type and evening type than in the morning type, while there was no difference in physical health-related quality of life among these groups. The percentages of the nursing students who have experienced absence, tardiness, falling asleep during class, and/or interference with academic achievement were higher in the evening type than in other groups.

**Conclusions:**

The present study has important implications for nursing students’ biological characteristics and lifestyle, which are associated with their health-related quality of life and academic performance.

**Supplementary Information:**

The online version contains supplementary material available at 10.1186/s12912-021-00748-3.

## Background

Sleep is important for maintaining and enhancing one’s health. Sleep patterns and sleep problems change along with the life-stage. Chronotype, the tendency towards morningness or eveningness underlying the circadian system, is influenced by age, gender [1], genetics [2], and external environment/circumstances including light exposure [3] or sociocultural conditions [4]. University students are likely to have activities and go to bed late at night due to their age-dependent delayed endogenous circadian clock (i.e., late chronotype) [1], school work, extracurricular activities, club activities, or part-time jobs. Nevertheless, they are forced to wake up early controlled by the social clocks (e.g., early-morning classes or early-morning practice in club activities). Therefore, late chronotype and resulting insufficient sleep are typical sleep problems in this age group. Previous studies have reported that these sleep problems are negatively associated with mental health [5], health-related quality of life [6, 7], and academic performance [8, 9] in the young population. Specifically, late chronotypes are often accompanied by unique eating behaviors (e.g., breakfast skipping and evening energy intake) [10, 11], which result in metabolic disorders including obesity and diabetes [12, 13]. In addition, late chronotypes are associated with poor mental health [14, 15] and poor academic achievement in university students [16]. Thus, being a late chronotype should be regarded as a risk factor for health problems and daytime functioning in this age group.

Sleep-related problems are not rare in nursing students: 9–12% have difficulty initiating sleep, 8–11% have difficulty maintaining sleep, and 8–9% have early morning awakening [17, 18]. Furthermore, the prevalence of insomnia, sleep disturbance, or poor sleep ranges from 19 to 56% [17–19]. Average sleep duration in nursing students varies among countries: 7 h in Turkey [20] and 5 h in Philippines [21], with regional differences in sleep duration among university students in general [22]. The percentage of nursing students who sleep less than 7 h was reported as 61% in an Italian university [17], 34% in a Scottish university [23] and 70% in a Turkish university [19]. In a previous study on daytime sleepiness in nursing students, 11% of the participants had daytime sleepiness, which was associated with insomnia symptoms, second year students and living alone [24]. In a study conducted in a school of nursing in Indonesia, 24.3% of male nursing students and 28.8% of female nursing students had daytime sleepiness [25]. In another study conducted in Taiwan, 35% of the first-year nursing students experienced daytime sleepiness at the beginning of the semester [26]. Taken together, more than a few nursing students may experience sleep problems, including insomnia, insufficient sleep, and daytime sleepiness. Furthermore, the previous studies reported that insufficient sleep worsened mental health [27] and health-related quality of life [21], that daytime sleepiness interfered with academic achievement [24, 28], and that subjective poor sleep quality was associated with both physical and mental health [29, 30] in nursing students. Nursing students have more stress, anxiety, sleep disturbance, and have more stress-related illness than general undergraduate students [31, 32] partially due to great amount of academic work [33]. Therefore, misalignment between their internal clocks and external environment should be taken into consideration for their physical/mental health and daytime functioning, including academic achievement. Some previous studies recommend stress coping strategies as an approach to address sleep problems [29, 34]; however, to our knowledge, no studies have focused on nursing students’ sleep and daily functioning considering their chronotype.

The purpose of the present study is to clarify chronotype and its association with lifestyle, health-related quality of life, and academic performance among nursing students. Building healthy lifestyles during their school days may contribute to improving not only their physical/mental health but also the quality of medical services (e.g., prevention of medical accidents, lowering the turnover rate of nurses) in the future.

## Methods

### Participants and data collection

This cross-sectional study was conducted at six universities with nursing courses in Japan between June 2018 and October 2019. Except for a university in the Tohoku region, a northern area of Japan (*n* = 57), all the universities were located in the Kanto region (i.e., Tokyo, Kanagawa, Chiba), urban areas in Japan. Self-reported questionnaires were distributed to undergraduate nursing students during the term of normal classes, not in the practical training period. From the questionnaire, we obtained the student IDs of all participants to enable us to provide feedback to those students who wanted to know about their individual results. We distributed the questionnaires during classes at each university and collected them using a collection box outside the classrooms or by having the participants return them in sealed envelopes after class to keep their responses private. We assured the participants that their participation was voluntary, and their participation/nonparticipation could have no educational disadvantage because the research collaborators (each university’s lecturers) had no access to the participants’ identities. Both the questionnaire collection and the return of individual feedback were handled using a collection box or a sealed envelope. We returned the sealed envelopes personally to those students who wanted feedback on their results; students who preferred not to receive the results directly from university lecturers could opt to receive them via postal mail or email. We only used their personal information (i.e., name, address, email address) to return the feedback results. We conducted these processes in accordance with the administrative regulation of each university’s ethics approval committee. The numbers of questionnaires distributed were 1123 (freshman: *n* = 231; sophomores: *n* = 435; juniors: *n* = 268; seniors: *n* = 189). We received 519 completed questionnaires. Because the number of male students is small (7%) and their chronotype differs from female students, we analyzed only the data obtained from female nursing students. Thirty-eight respondents were excluded from subsequent analyses because their chronotype could not be determined due to missing data. Finally, 447 valid data were used for analyses in the present study. This manuscript was written in accordance with the STROBE statement for cross-sectional studies (Additional file 1).

### Measures

The questionnaire was written in Japanese and consisted of the following items (Additional file 2).

#### Characteristics of nursing students and lifestyle/social activities

Students were required to answer questions on age, sex, grade, starting and closing time of class, whether they were living alone or not, commute time, exercise habits per week (≥ 4 days /2–3 days/ ≤ 1 day/ none), duration of habitual exercise, start time of exercise, engagement in part-time job (yes /no), part-time job after 10 p.m., starting and closing time of part-time job, participation in a club activity (yes/no), and starting and closing time of club activity. Frequency of exercise habit was converted into a binary outcome (0: none, 1: ≥ 4 days /2–3 days/ ≤ 1 day) for analysis.

Data on sleep pattern included information on sleep duration, bedtime, awakening time on weekdays and on weekends. The Japanese version of Epworth Sleepiness Scale (ESS) (Cronbach’s α = 0.88) [35–37] was used to assess the level of daytime sleepiness. The total sum score ranges from 0 to 24, and ≥ 11 is regarded as having excessive daytime sleepiness. Sleep quality was assessed with the Japanese version of Athens Insomnia Scale (AIS) (Cronbach’s α = 0.89) [38, 39], which indicates possible insomnia when one has a score above five out of the total score. In order to assess sleep-related symptoms, the self-report questionnaire included questions on difficulty initiating sleep, difficulty maintaining sleep, early morning awakening, and/or difficulty awakening.

Information regarding eating patterns included meal time, duration of meal, skipping meals, and frequency of skipping meals at breakfast, lunch, and dinner. In addition, students were required to answer questions on body weight gain after entering the university. Body mass index (BMI) was calculated by self-reported height and weight.

#### Chronotype

To assess chronotype, the Japanese version of Morningness-Eveningness Questionnaire (MEQ) (Cronbach’s α = 0.84) [40–42] was used. This questionnaire consists of 19 items, and a total score below 42 is defined as evening type, 42–58 as intermediate type, and above 58 as morning type.

#### Health-related quality of life and academic performance

For evaluating health-related quality of life, the physical component summary score and mental component summary score of health-related quality of life by 8-item short form (SF-8) Health Survey [43–45] was used. These scores were standardized with a mean of 50.

The questionnaire included questions on frequency of absence, tardiness, and falling asleep during class (often/sometimes/none) and interference with academic achievement (yes/no). Frequency was converted into a binary outcome (0: none, 1: often/sometimes).

### Statistical analyses

Representative values were shown as mean ± standard deviation. Kruskal-Wallis tests and Chi-square tests followed by residual analyses were performed for continuous variables and categorical variables, respectively. To identify the related factors with chronotype, the generalized linear mixed effect model was used. In the model, explanatory variables; age, living alone, start and closing time of class, commuting time, excise habit, part-time job and club activity were set as fixed effect, and university and grade were set as random effect. In the analysis of generalized linear mixed effect model, the degree of association was represented as adjusted odds ratio and 95% confidence interval. In these analyses, complete-case analysis was performed, but no imputation for missing data was performed because missing data were only 12% of the total. The significance level was defined as *p* < 0.05. Generalized linear mixed effect model was analyzed using R statistical software version 3.5.1 (R Core Team, Vienna, Austria). “lme4” package [46], and other analysis were performed by IBM SPSS Statistics 25 (IBM Corporation, Armonk, USA).

### Ethics

Ethical approval for this survey was granted by the Ethical Committee of the Institute of Neuropsychiatry (No.162). Six universities cooperated with permission of the ethical approval committees; two universities were approved by the ethical committee of the particular university (Teikyo University and Shonan University of Medical Sciences), and the ethical committees of the other four universities delegated ethical approval to the Institute of Neuropsychiatry. All the subjects participated on a voluntary basis, and responses were treated anonymously. Written informed consent was obtained from all participants.

## Results

### Reliability of the validated scales

This study’s Cronbach’s alpha values were 0.783 for the AIS, 0.779 for the ESS, 0.812 for the SF-8 Health Survey, and 0.708 for the MEQ.

### Chronotype and lifestyle/social activities in nursing students

The number of evening type individuals was 80 (17.9%), while those of morning type and intermediate type were 48 (10.7%) and 319 (71.4%), respectively.

The valid response rate for each grade was as follows: freshman, 66.2%; sophomore, 38.9%; junior, 27.6%; and senior, 27.0%. As for the chronotype, no difference was found among the grade groups (*p* = 0.200). Characteristics of nursing students and lifestyle/social activities for each chronotype were shown in Table 1. Students ranged in age from 18 to 27, and there was no difference in age among the three chronotype groups. Among the students with evening type, the percentages of the students who lived alone, those who had part-time job, or those who engaged in part-time job after 10 p.m. were higher, and the percentage of the students who had an excise habit and/or club activity was lower than that of the morning type and intermediate type. Frequency of part-time job was lower, and starting / closing time of part-time job was earlier in the morning type than in the intermediate type and evening type. In the generalized linear mixed effect model analysis, evening type was associated with living alone (adjusted odds ratio: 2.6, 95% confidence interval: 1.2–5.5), part-time job (adjusted odds ratio: 3.5, 95% confidence interval: 1.3–9.3), and club activity (adjusted odds ratio: 0.3. 95% confidence interval: 0.2–0.7) (Table 2).

**Table 1:** Characteristics of nursing students and lifestyle/social activities

	Morning type (*N* = 48)		Intermediate type (*N* = 319)		Evening type (*N* = 80)	
Age (y.o)	20.2 ± 1.5		19.7 ± 1.2		19.7 ± 1.1	
Living alone (%)	9		18		30	*
Start time of class	9:13 ± 0:57		9:09 ± 0:54		9:13 ± 0:55	
Closing time of class	17:04 ± 1:19		16:45 ± 1:27		16:40 ± 1:15	
Commuting time (min)	68.5 ± 30.0		61.7 ± 31.4		59.4 ± 33.6	
Excise habits (%)	54		43		30	*
Duration of exercise (min)	109.2 ± 108.5		91.0 ± 66.6		85.7 ± 62.1	
Start time of exercise	16:28 ± 4:43		15:54 ± 4:12		18:01 ± 3:11	
Part-time job (%)	75		79		89	*
Part-time job after 10:00 PM (%)	13	*	23		38	*
Frequency of part-time job ^a^ (per week)	2.1 ± 1.0	†, ‡	2.6 ± 1.1		2.6 ± 1.1	
Start time of part time job	13:37 ± 4:39	†, ‡	14:47 ± 3:28		16:25 ± 3:27	
Closing time of part time job	19:05 ± 4:29	†, ‡	21:31 ± 3:00		22:12 ± 3:10	
Club activity (%)	41		35		19	*
Frequency of club activity (per week)	1.7 ± 1.0		1.7 ± 1.0		1.5 ± 0.7	
Start time of club activity	17:10 ± 1:35		17:22 ± 1:25		17:09 ± 2:28	
Closing time of club activity	20:00 ± 1:22		19:53 ± 1:26		19:37 ± 2:15	

mean ± standard deviation, † vs Intermediate (*P* < 0.05), ‡ vs Evening (*P* < 0.05),

*|adjusted standardized residual|≧ 1.96,

^a^average of the students who answered having a part-time job

**Table 2:** Factors associated with evening type by the generalized linear mixed effect model

Predictors	Odds ratio	95% confidence interval	*P* value
Age	0.9	0.7–1.2	0.669
Living alone	2.6	1.2–5.5	0.011^*^
Start time of class	1.1	0.8–1.5	0.654
Closing time of class	1.0	0.8–1.3	0.886
Commuting time	1.0	1.00–1.02	0.258
Excise habit	0.8	0.4–1.5	0.501
Part time job	3.5	1.3–9.3	0.013^*^
Club activity	0.3	0.2–0.7	0.004^*^
**Random Effects**		**Variance**	
University (*n* = 6)		0.0	
Grade (*n* = 4)		0.0	

* *P* < 0.05

### Chronotype and sleep pattern

Sleep patterns in each chronotype were shown in Table 3. Bedtime and wake-up time on both weekdays and weekends were the latest in the evening type. Sleep duration on weekdays was shorter in the evening type (5.2 h) than in the morning type (6.2 h) and in the intermediate type (5.8 h). The AIS score was the highest in the evening type, however there was no difference in daytime sleepiness (ESS score) among chronotype groups. Among those identified as evening type, no students had a symptom of early morning awakening, while 19% of students had these symptoms in the morning type. The prevalence of difficulty awakening was higher in the evening type (70%) than in the morning type (15%).

**Table 3:** Chronotype and sleep pattern/sleep-related symptoms

	Morning type (*N* = 48)		Intermediate type (*N* = 319)		Evening type (*N* = 80)	
Bedtime on weekdays	23:36 ± 0:52	†, ‡	0:30 ± 1:01	‡	1:09 ± 1:21	
Wake-up time on weekdays	5:56 ± 0:48	†, ‡	6:27 ± 0:46	‡	6:55 ± 2:50	
Sleep duration on weekdays (h)	6.2 ± 0.9	‡	5.8 ± 1.1	‡	5.2 ± 1.3	
Bedtime on weekends	23:31 ± 1:04	†, ‡	0:43 ± 1:20	‡	1:34 ± 1:26	
Wake-up time on weekends	7:04 ± 1:09	†, ‡	8:49 ± 1:37	‡	10:11 ± 1:54	
Sleep duration on weekends (h)	7.3 ± 1.2	†, ‡	8.0 ± 1.6		8.3 ± 1.9	
Athene Insomnia Scale score	4.4 ± 3.1	†, ‡	6.1 ± 3.7	‡	7.4 ± 3.7	
Epworth Sleepiness Scale score	10.3 ± 4.9		11.7 ± 4.7		12.4 ± 5.4	
Difficulty initiating sleep (%)	15		27		31	
Difficulty maintaining sleep (%)	15		9		13	
Early morning awakening (%)	19	*	5		0	*
Difficulty awakening (%)	15	*	46		70	*

mean ± standard deviation, † vs Intermediate (*P* < 0.05), ‡ vs Evening (*P* < 0.05),

*|adjusted standardized residual|≧1.96

### Chronotype and eating pattern

Eating patterns in each chronotype were shown in Table 4. Breakfast and dinner time were the earliest in morning type. At breakfast, evening type had the shortest meal time. At every meal, the percentages of skipping meals were the highest (65% for breakfast, 21% for lunch, and 27% for dinner) in the evening type than in other chronotypes. Although there was no difference in BMI among chronotype groups, the percentage of the students who gained body weight after entering university was higher in the evening type (45%) than in the intermediate type (29%) and in the morning type (35%).

**Table 4:** Chronotype and eating pattern / body weight change

	Morning type (*N* = 48)		Intermediate type (*N* = 319)		Evening type (*N* = 80)	
Breakfast time	6:23 ± 0:42	†, ‡	6:51 ± 0:49		6:52 ± 0:47	
Meal time of breakfast (min)	20.0 ± 7.8	†, ‡	16.1 ± 8.1	‡	12.8 ± 7.3	
Skipping breakfast (%)	19	*	38		65	*
Frequency of skipping breakfast ^a^ (per week)	2.6 ± 1.6		2.8 ± 1.6	‡	3.8 ± 1.9	
Lunch time	12:04 ± 0:15		12:07 ± 0:18		12:06 ± 0:20	
Meal time of lunch (min)	26.8 ± 10.3		24.3 ± 9.3		25.8 ± 9.8	
Skipping lunch (%)	10		7	*	21	*
Frequency of skipping lunch ^a^ (per week)	1.6 ± 0.9		2.3 ± 1.6		2.9 ± 1.3	
Dinner time	19:07 ± 1:05	†, ‡	19:30 ± 1:32		19:43 ± 1:12	
Meal time of dinner (min)	34.3 ± 11.9		31.1 ± 15.3		33.0 ± 15.0	
Skipping dinner (%)	13		14	*	27	*
Frequency of skipping dinner ^a^ (per week)	4.0 ± 2.3		2.6 ± 1.2		2.4 ± 1.4	
Body mass index	20.4 ± 2.1		20.6 ± 3.1		20.2 ± 2.5	
Body weight gain (%)	35		29	*	45	*

mean ± standard deviation, † vs Intermediate (*P* < 0.05), ‡ vs Evening (*P* < 0.05),

*|adjusted standardized residual|≧1.96,

^a^average of the students who answered having skipped meals

### Chronotype and health-related quality of life / academic performance

Mental health-related quality of life was lower in the intermediate type and evening type than in the morning type, while there was no difference in physical health-related quality of life among chronotype groups (Table 5).

**Table 5:** Chronotype and health-related quality of life / academic performance

	Morning type (*N* = 48)		Intermediate type (*N* = 319)		Evening type (*N* = 80)	
Health-related quality of life: Physical component summary score	51.4 ± 5.1		51.1 ± 5.3		50.6 ± 6.1	
Health-related quality of life: Mental component summary score	50.0 ± 6.4	†, ‡	46.3 ± 8.1		46.2 ± 8.5	
Absence (%)	0	*	18	*	43	*
Tardiness (%)	9	*	26	*	55	*
Falling asleep during class (%)	78	*	89		97	*
Interference with academic achievement (%)	14	*	27		45	*

mean ± standard deviation, † vs Intermediate (*P* < 0.05), ‡ vs Evening (*P* < 0.05),

*|adjusted standardized residual|≧1.96

The percentages of the nursing students who have experienced absence, tardiness, falling asleep during class, and/or interference with academic achievement were higher in the evening type than in other chronotypes (Table 5).

## Discussion

### Chronotype and lifestyle/social activities in nursing students

The present study revealed that as high as 18% of the participants were identified as evening type. Furthermore, living alone, engaging in a part-time job and/or club activity were associated with evening type after adjusting for confounding factors. The students who live alone can make their life schedule themselves, independent of their family, which promotes delay of circadian rhythm. On the contrary, engagement in a club activity may restrict and properly regulate students’ life schedule. Among the students with evening type, as high as 89% of them had a part-time job. In addition, 38% of them were engaged in a part-time job after 10 p.m. The adjusted odds ratio of having a part-time job on evening type was 3.5 (95% confidence interval: 1.3–9.3), confirming the association between a part-time job and a later chronotype. The results of the association between a late chronotype and living alone, club activity, or part-time job suggest that lifestyle approaches would be helpful to prevent worsening delayed sleep–wake rhythms in nursing students.

It is noteworthy that a number of participants skipped breakfast, which was associated with a late chronotype. Many previous studies have reported an association between skipping breakfast and evening type [10, 47, 48]. Skipping breakfast is unhealthy eating behavior which contributes to metabolic disorders, including obesity and diabetes [49, 50]. In the present study, skipping lunch and dinner was associated with chronotype as well as skipping breakfast. This may suggest that their dietary schedule regulated by circadian rhythm is collapsed as well as their sleep-wake schedule. The percentage of the students who gained weight after entering university was higher in the evening type, although there was no difference in BMI among chronotypes. According to previous studies, chronotype is associated with food preference/intake; evening type individuals had a higher intake of energy, sucrose, fat, and saturated fatty acids than morning type individuals [11]. In contrast, morning type individuals had a higher intake of fruit [51]. Although information regarding food intake could not be obtained in the present study, a preference for unhealthy food intake may cause weight gain after nursing students with evening type enroll in university. Even though nursing students study the importance of nutrition, sleep, and physical activity for health, they are required to understand their biological preference for delayed circadian rhythms and the importance of regular food intake. Furthermore, knowledge of sleep disorders including insomnia, circadian rhythm disorder, and hypersomnia needs to be disseminated to nursing students and nursing teachers. Previous studies have reported that 9–12% of nursing students had difficulty initiating sleep [17, 18], and their sleep duration differed by country [17, 19–23]. In the present study, one-third of the students with evening type had difficulty initiating sleep even though their sleep duration was insufficient (5.2 h on weekdays). This is because they suffer from insomnia and/or their endogenously delayed circadian rhythm disturbs the process of falling asleep. For nursing students, maintaining sleep–wake rhythm is important for a healthy lifestyle, including sleep [52] and eating habits [53]. Severely delayed sleep–wake rhythm, which is diagnosed as delayed sleep–wake phase disorder in a clinical setting, could also disrupt nursing students’ social lives [54]. A medical approach is needed in the case that a sleep problem cannot be solved by a student’s own effort.

### Chronotype and health-related quality of life / academic performance

Previous studies have shown that insufficient sleep, daytime sleepiness, and poor sleep were associated with poor mental health, lower health-related quality of life, and lower academic achievement [21, 24, 27–30]. The present study revealed that chronotype was associated with mental health-related quality of life, but not with physical health-related quality of life. In mental health-related quality of life, the score was lower in the evening type and intermediate type than in the morning type. In middle-aged or older adults, late chronotype was associated with poor physical health-related quality of life [7]. However, in the young population, the impact of late chronotype on physical health-related quality of life was not apparent. It can be considered that long-term late sleep-wake rhythm and following insufficient sleep bring about future lifestyle-related disease occurring after middle age.

Regarding academic performance, more than half of the evening type nursing students experienced absences and tardiness, and almost all the students had experience falling asleep during class. In Japan, nursing students face busy schedules due to a challenging curriculum consisting of practical training, study for registered nurse licensure exams, and completion of a graduation thesis. Their busy schedules accelerate late chronotype and cause their physical fatigue [55]. Thus, not only nursing students but also nurse teachers, who need to manage students’ health problems and academic achievement, must understand that their biological characteristics (i.e., delayed sleep–wake rhythm) underlie these problems. One Canadian study reported that circadian rhythm disruption by shift work was related to nursing fatigue, which can pose patient safety risks, including medication errors [56]. Accumulating evidence of nursing students’ biological characteristics and early education on the importance of the sleep–wake rhythm could promote nurses’ health maintenance, thereby improving their turnover rate and the quality of nursing care. Considering the association between the chronotype and higher levels of depressive symptoms among nurses [57], establishing a healthy lifestyle to prevent a delayed sleep–wake rhythm would contribute to their future health and work performance as a nurse.

### Limitations

The present study had some limitations. First, the sample suffered from a weak representativity of the study population since the targeted nursing universities were not selected randomly from areas throughout Japan. All the nursing universities that collaborated in this survey were located in urban areas. Thus, further investigation on nursing students in rural area is needed before the results can be generalized. Second, the survey was conducted during the course of normal classes. According to some previous studies, nursing students experience stress during practical training [58, 59]. During practical training, their sleep patterns and the effect of chronotype on health-related quality of life–academic performance may differ from the results of this study. This study’s valid response rate was higher among the freshman than the other grades, but the freshmen had not yet received practical training. Thus, a comparison of the relationship between sleep patterns and daytime performance among the grades should be investigated. Third, the causal relationship between chronotype and outcomes could not be ascertained in this cross-sectional study. Finally, we analyzed data from only female nursing students. Eveningness is more severe in men than in women [1]; therefore, the impact of chronotype on health-related quality of life–school performance in male nursing students may differ from that of female students. Further longitudinal studies including a sleep survey during practical training in both male and female nursing students are needed in the future.

## Conclusions

The present study assessed chronotypes and their associated factors in nursing students. Late chronotype was associated with living alone, having a part-time job, engaging in club activities, and an unhealthy lifestyle, including delayed sleep-wake phase, insufficient sleep, and skipping of meals. The students with evening type and intermediate type had poor mental health-related quality of life than those with morning type. Most of the evening type nursing students experienced absences, tardiness, and falling asleep during class. The present study has important implications for nursing students’ biological characteristics and lifestyle, which are associated with their health-related quality of life and academic performance. Accumulating evidence of nursing students’ biological characteristics could help promote their health maintenance, thereby improving the nursing turnover rate and care quality.

## Supplementary Information

**Additional file 1.** STROBE checklist

**Additional file 2.** Questionnaire

## Data Availability

The datasets used and/or analyzed during the current study are available from the corresponding author on reasonable request.

## Abbreviations

AIS: Athens Insomnia Scale
BMI: Body Mass Index
ESS: Epworth Sleepiness Scale
MEQ: Morningness-Eveningness Questionnaire
SF-8: 8-item Short Form
