# Supplementary material for: Sleep–wake rhythm and its association with lifestyle, health-related quality of life and academic performance among Japanese nursing students: a cross-sectional study
Source: BMC Nurs. 2021 Nov 9;20:225. doi: 10.1186/s12912-021-00748-3 (PMC8576865; doi:10.1186/s12912-021-00748-3)
Supplement: Supplementary file 2 — Additional file 2. Questionnaire [file 12912_2021_748_MOESM2_ESM.docx]

Appendix 2. Question items

| Original questions about socio-demographic characteristics, lifestyle/social activities and daytime dysfunction in school life |
| --- |
| 1. Age (years old)  2. Sex (1: male, 2: female)  3. Grade (1: freshmen, 2: sophomores, 3: juniors, 4: seniors)  4. Start and end times of class (hour)  5. “Do you currently live alone?” (1: yes, 2: no)  6. One-way commute time (minutes)  7. “Please choose that, which describes your exercise habit”  (1: ≥4 days/week, 2: 2-3 days/week, 3: ≤1 days/week, 4: not at all)  8. Duration of habitual exercise (minutes)  9. Start time of exercise  10. “Do you have a part-time job?” (1: yes, 2: no)  11. Starting and closing time of part-time job  12. “Do you participate in a club activity?” (1: yes, 2: no)  13. Starting and closing time of club activity  14. Sleep duration (hours), bedtime and awakening time on weekdays and on weekends  15. “Do you have any problems during nocturnal sleep?” (multiple answers) ( difficulty initiating sleep, difficulty maintaining sleep, early morning awakening, difficulty awakening)  16. Meal time (breakfast, lunch and dinner)  17. Duration of meal (breakfast, lunch and dinner)  18. Skipping meals (1:yes, 2:no) and frequency per a week (breakfast, lunch, dinner)  19. Body weight gain after entering the university (1:yes, 2:no)  20. Height (cm) and weight (kg)  21. “Have you missed class, training, or extracurricular activities due to sleep?” (1: none, 2: sometimes, 3: often)  22. “Have you been late for class, training, or extracurricular activities due to sleep?” (1: none, 2: sometimes, 3: often)  23. “Have you fallen asleep during class?” (1: none, 2: sometimes, 3: often)  24. “Have you experienced interference with your academic performance (attendance at class, training, extracurricular activities, homework, examinations, etc.) due to sleep?” (1: no, 2: yes) |
| Epworth Sleepiness Scale (8 items) ^35, 36)^ |
| Athens Insomnia Scale (8 items) ^37,38)^ |
| Morningness-Eveningness Questionnaire (19 items) ^39,40)^ |
| SF-8 Health Survey (8 items) ^41,42)^ |
